# Hemangioma: Recent Advances

**DOI:** 10.12688/f1000research.20152.1

**Published:** 2019-11-18

**Authors:** Austin DeHart, Gresham Richter

**Affiliations:** 1 Otolaryngology, Head and Neck Surgery, Arkansas Children's Hospital at the University of Arkansas for Medical Science, Little Rock, Arkansas, 72202, USA

**Keywords:** Hemangioma, vascular anomaly

## Abstract

Hemangiomas are common benign vascular tumors that often present in childhood. Diagnosis is based on clinical history, physical examination, and, when unclear, assisted with ultrasound or MRI. While the majority are small, nonproblematic, and can be managed conservatively, some hemangiomas may be associated with underlying syndromes or concerning for visceral involvement. Symptomatic lesions may develop ulceration, bleeding, vision disturbances, functional limitations, or disfigurement. The ideal treatment for a symptomatic hemangioma is often multimodal and may vary depending on the size, location, and proximity to critical structures. Medical treatments include topical beta blockers, oral propranolol, or steroid injections. Surgical resection and laser therapies may be necessary to optimize long term outcomes.

## Introduction

Infantile hemangiomas (IHs) are benign vascular tumors that are common in children. They are present in an estimated 5% of the population and are characterized by abnormal proliferation of endothelial cells and abnormal blood vessel structure ^1^. While most IHs are sporadic, some familial clustering does occur ^2^. Other risk factors include first trimester bleeding, preeclampsia, prematurity, advanced maternal age, placental abnormalities, female gender, and low birth weight ^2^.

## Classification

**Figure 1.**
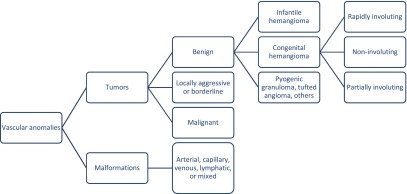
Abbreviated International Society for the Study of Vascular Anomalies (ISSVA) classification system for vascular anomalies. Adapted from the ISSVA Classification of Vascular Anomalies ©2018 International Society for the Study of Vascular Anomalies. Available at https://www.issva.org/classification. Accessed 16 March 2018 ^3^.

Hemangiomas are a tumor subtype of vascular anomalies as classified by the International Society for the Study of Vascular Anomalies (ISSVA), shown in Figure 1, and can be further described as either infantile or congenital ^3^. IHs are absent, subtle, or very small at birth, become clinically evident around 1 month of age, and have rapid growth during the first 6 months of life. They stabilize in growth at around 7–12 months of age and subsequently, and uniquely, undergo involution in early childhood. The rate of involution varies from one hemangioma to another, even in the same patient with more than one. IHs express GLUT1, a receptor also found on placental blood vessels, which has led to a theory that they may be caused by abnormal implantation of progenitor cells disrupted from the placenta during fetal development ^4^.

Congenital hemangiomas (CHs) are present at birth at their maximal size and lack a growth phase ^5^. They are categorized as rapidly involuting CH (RICH), non-involuting CH (NICH), or partially involuting CH (PICH) depending on their clinical behavior. RICHs have been reported to develop transient mild-to-moderate thrombocytopenia and consumptive coagulopathy as they involute ^6^. CHs do not exhibit GLUT1 staining ^7^.

IHs are further named based on the depth of growth as superficial, deep, or mixed (superficial and deep) or may exhibit reticular, abortive, or minimal growth. IHs can also be focal, multifocal, or segmental based on their anatomic distribution. Focal, or localized, hemangiomas are discrete, solitary lesions with well-defined borders. Multifocal hemangiomas are discrete lesions that occur at multiple sites. Segmental hemangiomas are larger plaque-like lesions that may follow dermatomal distributions, often develop ulcerative complications, and demonstrate prolonged proliferation and involution phases. They are also associated with syndromes that require additional work-up.

## Diagnosis

A thorough clinical history and physical examination leads to the accurate diagnosis of hemangiomas. Key aspects of the history include onset, timing, and progression of lesion growth, ulceration, bleeding, vision disturbance, and impact on function. Routine imaging is not necessary but can be useful in deeper lesions and diagnostic challenges.

Ultrasound of IH is inexpensive, fast, and convenient. IH will demonstrate a well-defined mass with high vessel density, without abnormality of the surrounding fat, with uniform, pulsatile, fast-flow vascularity on Doppler with arterial and venous waveforms ^8^. MRI can be used to provide further information regarding the extent of involvement in local tissue for deep disease. IHs are hyperintense on T2 and isointense on T1, with intense post-contrast enhancement on MRI. RICH and NICH may have more variability and heterogeneity in their imaging characteristics compared to IH on both ultrasound and MRI ^8^.

Imaging with a screening abdominal ultrasound is recommended for children with five or more multifocal CHs because of an increased risk of concurrent liver hemangioma ^9^. Multifocal and diffuse hepatic hemangiomas can lead to serious and life-threatening complications, so early identification is important and, when present, examination of thyroid function should be considered.

While superficial cutaneous lesions can be easily diagnosed on exam and history, deep hemangiomas may pose more of a challenge. Parotid hemangiomas are the most common parotid neoplasm in children and can present in patients younger than 4 months of age with a deep palpable cheek mass without cutaneous manifestations ^10^. Ultrasound is useful to confirm diagnosis and is often sufficient as a singular imaging modality. MRI or immunohistology can be used if diagnostic uncertainty remains ^10^.

## Associated findings and syndromes

Rarely, hemangiomas can be associated with other congenital anomalies. Segmental hemangiomas in a beard distribution over the chin and neck have increased risk (60%) of concurrent subglottic hemangioma ^11^. PHACE syndrome, the constellation of posterior fossa anomalies, hemangioma, arterial lesions, cardiac abnormalities or coarctation of the aorta, and eye abnormalities, is associated with large segmental IH of the face, scalp, and neck ^12^. Complete screening should be considered in patients with large segmental hemangiomas of the head or central trunk. Patients with PHACE may be at increased risk of arterial ischemic stroke depending on their individual vascular anatomy ^13^. Screening for PHACE should include physical exam, echocardiogram, MRI/MRA of the head and neck, and an ophthalmology exam ^12^. LUMBAR syndrome is a rare grouping of lower body IH, urogenital anomalies, ulceration, myelopathy, bony deformities, anorectal malformations, arterial anomalies, and renal anomalies ^14^. This also is described using the acronyms SACRAL, for spinal dysraphism, anogenital anomalies, cutaneous anomalies, renal anomalies, and lumbar hemangioma, or PELVIS, for perineal hemangioma, external genital malformation, lipomyelomeningocele, vesicorenal abnormalities, imperforate anus, and skin tag ^15, 16^.

## Management

Owing to their natural tendency towards involution, most inconspicuous IHs can be managed with observation alone. IHs that require intervention include those that become symptomatic during the growth phase, with ulceration, bleeding, vision disturbance, and functional limitations (breathing), or when imminent disfigurement is expected. Historic and current treatment options include medical therapy (steroids/propranolol), surgical resection, laser therapy, or direct intralesional steroid injections. A combination of more than one treatment modality is often performed in larger or resistant lesions.

Propranolol is the mainstay of treatment for large or symptomatic IH, including subglottic and parotid disease. Typical dosing is 1–3 mg/kg/day divided into two to three doses ^5^. This is generally well-tolerated but may be associated with sleep disturbance, diarrhea, bronchial hyper-reactivity, and hypoglycemia. Dosing with feeds is recommended. It is contraindicated in patients with bradycardia, heart block, hypotension, and asthma ^5^. EKG prior to starting therapy should be considered. Infants younger than 8 weeks of age should be admitted for inpatient initiation of treatment for close monitoring ^17^. Early treatment during the proliferative phase is thought to be associated with improved outcomes ^18^. Over 90% of IHs respond to propranolol with reduction in size and color. Deeper components respond better than superficial ones. Residual disease may require laser or intralesional therapy or surgical interventions. Topical timolol is an alternative option to oral propranolol for smaller lesions and has shown promise in halting proliferation and inducing early involution when used for thin, superficial IH ^19^.

Corticosteroid therapy is an alternative option for patients who have contraindications or inadequate response to propranolol ^5^. Typical dosing is prednisone at 2–3 mg/kg daily and monitoring for side effects including adrenal axis suppression, cushingoid facies, irritability, and stomach irritation is recommended. For localized IH, intralesional steroid injection can be considered and can be used in conjunction with the favorable responses obtained with propranolol. Sirolimus may be a last-resort treatment in refractory cases.

Laser therapy is a treatment option typically used for persistent telangiectasia or residual lesions during or after hemangioma involution. Pulse dye laser is commonly used owing to its preferential absorption by hemoglobin; Nd:YAG is sometimes also used in lesions with significant venous drainage. Laser treatments during the growth phase of IH are controversial and generally not pursued because of the possibility of blistering, ulceration, and long-term pigmentation changes ^20^.

Surgical excision is reserved for ulcerative, bleeding, and significantly protruding hemangiomas. This can be performed alone or in combination with other treatments, especially when response to other treatments is limited or ineffective ^21^. Scarring from ulcerative hemangiomas often requires revision in combination with resection of residual disease. Delaying surgery until after involution allows for the excision of smaller and less vascular lesions. Hemangiomas with a significant vertical growth pattern are at higher risk of leaving undesirable fibrofatty residuum after involution, which can be addressed with surgical excision ^22^. This is particularly common in scalp IHs, which may develop alopecia during the involution phase and are easily excised with primary closure ^23^.

Hepatic hemangioma may require multimodal therapy, including treatment with steroids, propranolol, and embolization. Propranolol is currently favored owing to its low risk and high response rate. While not every hepatic hemangioma needs treatment, screening in these high-risk children has been shown to significantly reduce the risk of congestive heart failure, hypothyroidism, abdominal compartment syndrome, and mortality ^24^.

## Conclusion

Hemangiomas are congenital vascular lesions divided into congenital and infantile subtypes. CHs are rare and fully present at birth; usually treatment is not required. IHs are common childhood tumors that undergo a characteristic pattern of proliferative growth phase followed by slow involution. Symptomatic IHs can be treated with low-dose beta-blocker medication, with the best results occurring from initiation of treatment in the early phase. Multimodal therapy may be required in some cases involving a combination of laser, intralesional, medical, and surgical interventions. Residuum from IHs can be managed with laser treatment or surgical excision when needed.
